# Collective Impact through Public Health and Academic Partnerships: A Kentucky Public Health Accreditation Readiness Example

**DOI:** 10.3389/fpubh.2015.00044

**Published:** 2015-03-09

**Authors:** Angela L. Carman

**Affiliations:** ^1^Department of Health Management and Policy, College of Public Health, University of Kentucky, Lexington, KY, USA

**Keywords:** public health, workforce, academia, partnerships, collective impact

## Abstract

In the ever-changing, resource-limited public health environment, the use of partners found in the faculty and students of Colleges of Public Health can provide training, consultation, and technical assistance needed to increase local health department (LHD) workforce capacity to meet new public health demands including national public heath accreditation. This manuscript describes the provision of the backbone support activities of facilitation, data management, and project management by University of Kentucky’s College of Public Health to Kentucky’s LHDs seeking national public health accreditation.

## Introduction

Among the many recommendations for improvement of the public health system deemed “in disarray” in the 1988 Institute of Medicine (IOM) report, was a call for more formal interaction between public health academic settings and public health practice (1). Studies of the linkages between public health practice and schools of public health identify a variety of activities in existence between the two entities including public health practice steering/advisory committees in schools of public health, joint research opportunities, and the provision of technical assistance both from practice to schools and from schools to practice (2, 3). These activities and other partnership opportunities between academia and practice exist in formal agreements and in informal relationships (3).

Formal examples of partnerships include the Centers for Disease Control and Prevention (CDC)-funded Public Health Prevention Research Centers (PRC), which work as interdependent associations of accredited schools of public health, public health practice partners, and community members to conduct prevention research (4). In addition, HRSA-funded Public Health Training Centers (PHTC), first established in accredited schools of public health, assess the training needs of the public health workforce and deliver training to meet current and emerging public health needs of practicing public health workers (5, 6). Informal partnerships also exist and often arise from the interaction of individual faculty members in schools of public health and public health professionals who identify key activities where partnering together can meet the needs of both organizations (3).

The University of Kentucky’s College of Public health (UKCPH), site of both a PRC and PHTC, has a long history of partnership with the Kentucky governmental public health leadership and workforce. The governmental public health workforce in Kentucky includes workers located in both the Commonwealth of Kentucky Department of Public Health and in 61 local public health jurisdictions (15 multi-county districts and 46 single county health departments). Each of the 61 local public health jurisdictions vary greatly in size from city–county health departments with over 300 employees to small single county health departments with less than 10 employees (7).

Preparing the public health workforce to meet the ever-increasing demand for public health services in Kentucky is more important now, in the wake of an economic recession, than ever before. Kentucky, like many other states, experienced significant public health job loss and associated programmatic impacts during the 2008–2009 economic recession (8). This reduction in the workforce comes at a time when the need for preventive services, environmental services, and other public health initiatives continues to rise (8–10). The remaining members of the public health workforce attempt to provide services in an environment where efficiency, defined as “the use of minimal resources – raw materials, money, and people – to produce a desired volume of output” (11) is critical.

In this ever-changing, resource-limited public health environment, the use of partners to maximize the capacity of the governmental public health workforce becomes more important than ever (12). Colleges of Public Health such as UKCPH can provide links to other public health system partners and through training, consultation, and technical assistance increase on-the-ground local health department (LHD) capacity to meet new public health demands that include national public health accreditation. In this pilot study, UKCPH faculty and students developed a pilot tested elements of the Collective Impact model’s backbone support organization for public health accreditation readiness activities in partnership with Kentucky LHDs. This manuscript describes the local public health accreditation environment, the needs in Kentucky for accreditation readiness assistance, and the opportunity for collaboration between the UKCPH faculty and students and those health departments pursuing accreditation.

## Background/Literature Review

### National public health accreditation

In September 2011, the Public Health Accreditation Board (PHAB) launched the first voluntary accreditation system for state, local, and tribal public health agencies in the United States (13). This event is the result of years of effort that can be traced to a variety of watershed events in public health beginning with the 1988 IOM report, *The Future of Public Health*. In this report, the IOM characterized the United States public health as a “system in disarray” (1) indicating a great need for change and improvement. In 2002, IOM released another major report, The Future of Public Health in the Twenty-First Century, that made recommendations on a variety of public health issues including consideration of a public health accreditation system and increased training for public health leaders (14). In 2007, following recommendation by the exploring Accreditation Steering Committee, PHAB was incorporated (13).

The mission of PHAB is to promote and protect the health of communities by advancing the quality and performance of all public health departments in the United States (13, 15, 16). In 2009, PHAB conducted a national beta test in which 30 state, local, and tribal health departments, of varying sizes, completed the accreditation process and provided feedback on both the process and the accreditation standards. The goal of PHAB is to have 60% of the United States population served by an accredited health department by 2015 (13). Accreditation not only includes an emphasis on quality improvement, but creates focus, via the standards and measures for the 10 essential public health services (EPHS) (17). Specifically, PHAB places significant emphasis on community health assessments (CHAs) (EPHS #1), community health improvement plans (CHIPs) (EPHS #5), and agency strategic plans (EPHS #5) to require completion of these elements as prerequisites to applying for accreditation.

The CHA is defined by PHAB as

… a systematic examination of the health status indicators for a given population that is used to identify key problems and assets in a community. The ultimate goal of a CHA is to develop strategies to address the community’s health needs and identified issues. A variety of tools and processes may be used to conduct a CHA; the essential ingredients are community engagement and collaborative participation (18).

The need for LHDs to conduct a community needs assessment goes beyond the prerequisite requirement by PHAB. The CHA also provides the basis for the CHIP defined by PHAB as

A CHIP is a long-term, systematic effort to address public health problems on the basis of the results of CHA activities and the community health improvement process. This plan is used by health and other governmental education and human service agencies, in collaboration with community partners, to set priorities and coordinate and target resources (18).

In both the CHA and the CHIP, the LHD must evaluate the community and thus the partners and linkages within the public health system of that community (17). The remaining PHAB prerequisite, the agency strategic plan, sets the specific direction for the LHD (18). This direction may also be based on the results of the CHA and CHIP. For many LHDs, these processes are familiar, however, others, often due to the reduction in resources, have not completed a CHA or CHIP for many years or may not feel they have the expertise or staff to coordinate these processes.

### Community engagement

Of critical importance to the CHA and CHIP processes for LHDs is “community engagement and collaborative participation” (18). Scutchfield et al. substantiates this premise by stating that “community involvement is an absolute core value of effective public health practice” and “improving the public’s health demands citizens that feel connected to the decisions being made” (19). Kopell names this process “civic engagement,” which he defines as strengthening the relationship between the decision-makers and those affected by the decisions (12).

Local health departments that attempt to assess needs and develop and implement solutions to public health problems alone find that these solutions may be beyond the scope of a single governmental public health agency (20). However, inviting community members to the table to solve public health problems does not guarantee an effective outcome. Kopell terms a “faux civic engagement,” as an attempt to bring partners together in a process that is rushed and without the right people being involved. In “faux civic engagement” critical questions are not asked and participants feel that they have not been listened to, become frustrated, and wonder why they have wasted their time (12).

### Models for community engagement

A number of models for community engagement have been researched and implemented to provide structure to the process and increase the benefits of involving members of the community and specifically members of the public health system. Mobilizing for Action through Planning and Partnerships (MAPP) is a model developed by the National Association of County and City Health Officials (NACCHO) and the CDC. LHDs and their communities may use MAPP to conduct CHAs and CHIPs (21). MAPP includes organizing of community stakeholders, collaborative visioning, and community assessment using four tools with questions and strategic initiative development and actions (21).

Many additional models exist for providing structure to a CHA including healthy cities, with a focus on broad definitions of health, root causes, and system change (19). In addition, the Kettering Foundation has focused on citizens naming community problems as well as taking responsibility for solutions (22). Asset-mapping models are used by community members to identify and build upon work already in place in the community (23). Models may also be adopted from community-based participatory research such as Expanding the Empowerment Education Model (EEM), which utilizes listening, to internalize community positions, dialog, to discuss various community positions, deliberation, to reason and decide on a direction, and action, to implement community decisions (24). The Healthy Neighborhoods Initiative model utilizes quantitative information from statistics, qualitative information from interviews, and groups and asset-mapping strategies (25).

Understanding the necessity of collaboration to address large or small community issues is not a new concept. Regardless of the model selected to engage in assessing community needs or planning for improvement, a wide variety of progress levels emerge. Some groups are successful in obtaining community engagement and making a difference in the issue at hand while others flounder. Kania and Kramer propose not only a model for cross-sector coordination, Collective Impact, but within it provide an element unlike others previously mentioned – a supporting infrastructure. The authors propose “the expectation that collaboration can occur without a supporting infrastructure is one of the most frequent reasons why it fails” (26).

Kania and Kramer’s Collective Impact model specifies five conditions of community engagement success – having a common agenda, shared measurement systems, mutually reinforcing activities, continuous communication, and a backbone support organization (26, 27). The backbone support organization concept provides a supporting infrastructure for stakeholders engaged in CHA or community improvement planning through the provision of a dedicated staff separate from community partner organizations (26, 27). A specific Cincinnati-based education initiative, STRIVE, which utilizes the Collective Impact theory and employs the backbone support organization concept, synthesized backbone support duties down to facilitation, data management, and project management (26).

## Methods/Strategies/Intervention Applications

### Initial needs assessment

To provide focus to the UKCPH practice-based activities and in light of the Fall 2011 scheduled launch of public health accreditation, UKCPH faculty conducted a brief electronic survey of each LHD director in Kentucky regarding six elements of public health accreditation: identification of an accreditation coordinator (required by the PHAB process); completion within the last 3 years of the PHAB prerequisites – strategic plan, community needs assessment, and CHIP; completion of a quality improvement plan; and a process in place for updating and evaluating policies and procedures (see Table 1).

**Table 1:** **Accreditation readiness survey of Kentucky LHDs**.

Survey question	Yes	No	In process
Accreditation coordinator identified	29 (91%)	3 (9%)	0
Strategic plan completed	4 (12.5%)	15 (46.9%)	13 (40.6%)
Community health assessment completed	13 (40.6%)	7 (21.9%)	12 (37.5%)
Community health improvement plan completed	2 (6.3%)	19 (59.4%)	11 (34.4%)
Quality improvement plan	7 (21.9%)	14 (43.8%)	11 (34.4%)
Process for updating and evaluating policies and procedures	17 (53.1%)	8 (25%)	7 (21.9%)

Responses were received from 56% of the LHD directors in the Commonwealth of Kentucky. The majority of respondents had identified an accreditation coordinator (91%) and had a process in place for updating and evaluating policies and procedures (53.1%). However, although 40.6% of respondents had completed a CHA within 3 years, only 2% had completed a CHIP from the results of the needs assessment. Low responses were also received regarding the completion of a strategic plan (12.5%) and a quality improvement plan (21.9%).

University of Kentucky’s College of Public Health faculty, building from the Collective Impact model (26), the LHD director survey results, and the elements of a backbone support organization as identified by STRIVE (i.e., facilitation, data management, and project management) developed and pilot tested the elements of a backbone support organization as a role for academic public health to assist LHDs with accreditation readiness.

### Backbone support organization – duty 1 – facilitation

University of Kentucky’s College of Public Health faculty developed the Kentucky Accreditation Coordinator Learning Community, which consisted of LHD directors and accreditation coordinators interested in learning about the elements of accreditation. The group met monthly during which UKCPH faculty provided meeting management, agenda development, and training. Topics were introduced either by UKCPH faculty or by members of the group and included development of a web-based inventory of accreditation resources, development of an accreditation readiness team, partnership collaboration, strategic planning, use of public health students in data collection, development of a quality improvement programs, community needs assessments, and a variety of other issues.

Through monthly contact with LHD directors and accreditation coordinators via the accreditation learning community, UKCPH faculty fielded questions on a wide variety of accreditation readiness activities. One of the most frequent issues involved the need by LHDs for outside facilitation of community forums organized as a part of the CHA process. Using trained UKCPH facilitators, in January 2012, UKCPH faculty and students began a pilot test of community forum facilitation in a multi-county public health district in Kentucky. Facilitation of these forums included consultation with the management of the district on community forum agendas, stakeholder involvement, and process for obtaining community feedback based on the four assessments of the MAPP process.

### Backbone support organization – duty 2 – data management

As the pilot process evolved in the public health multi-county district, a need arose for data management to obtain a disease burden picture of each community for use in the Community Health Status assessment of the MAPP process. Members of the UKCPH faculty worked closely with the epidemiologist of the public health district to identify appropriate sources of information and develop a template for disease burden information which included county specific, state, and national information on social factors, maternal child health, behavior factors, diabetes indicators, access to care, cancers, and respiratory illness information. A sample of the format is provided in Table S1 in Supplementary Material.

Members of the UKCPH faculty also coordinated the use of public health masters and doctoral level students to take notes and provide meeting summaries following each community forum. Additional data management duties included development and analysis of electronic and paper community surveys for each county within the district, use of public health doctoral students and university medical librarians to identify evidence-based intervention information for community workgroups, and the collection of all information into an overall project report for community.

### Backbone support organization – duty 3 – project management

With lessons learned from the pilot project, UKCPH faculty members formalized the methodology used to facilitate community forums and provided data management to LHDs utilizing a project management flowchart. The format for delivery of project management services was based on community engagement theory and the MAPP process augmented by asset mapping, quality improvement, evidence-base intervention review, and measureable goals and objectives development (see Table 2).

**Table 2:** **Elements of the UKCPH accreditation readiness backbone support organization**.

Elements of a backbone support organization as identified by STRIVE (26)	UKCPH activities
Facilitation	Faculty helped develop the Kentucky Accreditation Learning Community
	Faculty taught sessions on accreditation readiness topics to the learning community participants
	Faculty facilitation of community forums for CHA/CHIP
Data management	Faculty and students collaborated with Kentucky regional epidemiologists to create a format and data sources from which a disease burden picture for LHD jurisdictions was created
	Faculty and students created community surveys and analysis of results for the CHA/CHIP process
Project management	Faculty and students provided technical assistance in the form of answering questions from LHDs, formalizing a process for CHA/CHIP facilitation and provided access to evidence-based interventions for LHDs to use in addressing identified health needs.

Continuing to serve as the backbone support organization to provide LHDs with resources and structure for community needs assessment and community health improvement planning (26, 27), UKCPH faculty members have provided all or part of this process and backbone support organization functions to additional counties in Kentucky. The goal for UKCPH’s process is to provide support such that any LHD willing to pursue accreditation can be assisted and moved forward in the process using these services.

## Discussion

In consideration of the increasing demands upon public health and the impact of reductions in funding on staff and programing, it is understandable that LHD staff might find CHA and community health improvement planning daunting tasks. Even though community engagement theories and models offer structure to bringing partners and stakeholders into the process of solving community problems, much of the work falls on a staff already burdened by day-to-day tasks and potentially unfamiliar with available models.

Employing the Collective Impact model for community engagement speaks to the essence of public health collaboration as it includes steps to provide a common agenda, measurements, and communication. However, this model also provides a role for public health academic partners – the role of the backbone support organization providing facilitation, data management, and project management for LHDs seeking national public health accreditation. Faculty can provide expertise in facilitation, data management, and project management to the LHD while including students in the deployment of these resources which provides a much needed practice-oriented learning experience. LHDs, however, must be aware that the responsibility for relationship building with community stakeholders is a component of CHA and community health improvement planning that cannot be outsourced to faculty or students in a backbone support organization. LHDs must be aware that the success of any community engagement process hinges on these relationships.

Working with members of Kentucky’s Accreditation Readiness Learning Community continues in topics such as facilitation, coalition building, team building, and quality improvement techniques. This learning community has enabled UKCPH to expand the reach of the backbone support organization and further the accreditation readiness and workforce capacity in Kentucky.

## Conclusion

The UKCPH process of becoming a backbone support organization for the LHDs of Kentucky seeking national public health accreditation was born from a desire to meet each organization “where they are” with regard to accreditation and to assist them to move forward. That desire and the interaction between UKCPH faculty members and LHD directors and accreditation coordinators through the Kentucky Accreditation Learning Community, in an environment impacted by reductions in staff and resources, produced a dynamic process that grew to meet additional needs with each application. For PRC’s, PHTC’s, Colleges of Public Health, or other organizations desiring to become backbone support organizations for the LHDs in their service areas, the following recommendations have been developed:

- Understand the environment in which LHD works – beginning with a needs assessment to identify an area where faculty and student resources can be useful.
- Increase the skills of the faculty and students to include facilitation, data management, and project management in order that the need for these backbone support organization services at the local level can be provided or supplemented.
- Base processes in proven methodology while remaining flexible enough to meet the LHDs “where they are.”

Providing a structure through the backbone support organization while removing some of the day-to-day burden of CHAs, and developing improvement planning through facilitation, data management, and project management will move the process of community engagement and community problem-solving forward. Consequently, Colleges of Public Health can assist public health practitioners and their partners to engage in the action steps of intervention and ultimately the improvement of the public’s health while providing real-world learning settings for students.

## Conflict of Interest Statement

The author declares that the research was conducted in the absence of any commercial or financial relationships that could be construed as a potential conflict of interest.

## Supplementary Material

The Supplementary Material for this article can be found online at http://www.frontiersin.org/Journal/10.3389/fpubh.2015.00044/abstract
