# Supplementary material for: Collective Impact through Public Health and Academic Partnerships: A Kentucky Public Health Accreditation Readiness Example
Source: Front Public Health. 2015 Mar 9;3:44. doi: 10.3389/fpubh.2015.00044 (PMC4353173; doi:10.3389/fpubh.2015.00044)
Supplement: Supplementary file 1 [file table_1.docx]

**Collective Impact through Public Health and Academic Partnerships: A Kentucky Public Health Accreditation Readiness Example**

Angela L. Carman, DrPH^1^

^1^Department of Health Management and Policy, University of Kentucky, Lexington, KY

**Correspondence:**

**Angela L. Carman, DrPH**Department of Health Management and Policy

College of Public Health

University of Kentucky

111 Washington Avenue, Suite 105C

Lexington, KY 40536-003 USA

[angela.carman@uky.edu](mailto:angela.carman@uky.edu)

**Supplementary Table S1:** Sample Data Management Format

| **Social Factors** | **County** | **State** | **US** | **Data Source** |
| --- | --- | --- | --- | --- |
| Population | 46,406 | 4,369,356 | 311,591,917 | US Census Bureau, 2011 |
| Race Stats White | 89.70% | 88.90% | 78.10% | US Census Bureau, 2011 |
| African American | 8.00% | 8.00% | 13.10% | US Census Bureau, 2011 |
| Hispanic | 1.90% | 3.20% | 16.70% | US Census Bureau, 2011 |
| % of high school graduates persons age 25+ | 83.50% | 81.00% | 85% | US Census Bureau, 2011 |
| % of persons below poverty level | 14.40% | 17.70% | 13.80% | US Census Bureau, 2011 |
| % of population over 5 language other than English spoken at home | 3.20% | 4.60% | 20.10% | US Census Bureau, 2011 |
| Self Rated Health Status (% of Adults who report fair or poor health) | 18% | 22% | - | County Health Rankings, 2012 |
| Children in single parent households | 34% | 32% | - | County Health Rankings, 2012 |
